# Supplementary material for: Distribution of trespass cannabis cultivation and its risk to sensitive forest predators in California and Southern Oregon
Source: PLoS One. 2021 Sep 1;16(9):e0256273. doi: 10.1371/journal.pone.0256273 (PMC8409643; doi:10.1371/journal.pone.0256273)
Supplement: Attachment — Submitted filename: Response to reviewers - Wengert et al..docx [file pone.0256273.s001.docx]

Greta M. Wengert

Integral Ecology Research Center

gwengert@iercecology.org

May 12, 2021

Dear Editors,

I have made substantial changes to this manuscript (“Distribution of trespass cannabis cultivation and its risk to sensitive forest predators in California and Southern Oregon”) in direct response to the Academic Editor’s and Reviewers’ comments. A concern of one of the reviewers was the focus on anticoagulant rodenticides and their presence at grow sites and subsequent risk to predators interacting with these sites. I added several citations and reports that directly document the presence of anticoagulant rodenticides and other pesticides at these sites. The manuscript already had several citations directly showing the mortality and exposure risk to 2 of the 3 species covered in this study, so I reinforced the link by highlighting the actual findings of these papers. I also completely reworked the Introduction at the suggestion of Reviewer 1, to focus less on the effects and presence of rodenticides, and more on the need to model cannabis distribution in relation to the ecology of the three species we focused on.

The Academic Editor inquired about the ability to share the data we used to build the analysis and model. The entire dataset of locations we used is law enforcement sensitive. Every grow site location is still considered a crime scene still under investigation by either California Department of Justice or United States Forest Service Law Enforcement and Investigations, and thus, we are not allowed to share these locations outside of law enforcement agencies. This is for public safety reasons and also to protect the investigations. We are legally bound within the agreements we have with different law enforcement agencies to not disclose these data outside of our organization (Integral Ecology Research Center).

I have also removed the reference to funding from the Acknowledgements section, and added a reference for logistical support on the part of the Forest Service. I have reworked the References section and the headings to match the formatting guidelines provided in the review.

I have not changed Figures 1, 2, 3, and 5 because they are not copyrighted. One of the authors (Rustigian-Romsos) created all those figures from publicly available spatial layers which are included at the end of this letter.

Finally, I have created a higher quality version of Figure 4 to meet quality requirements.

I believe we have adequately addressed all the concerns raised by Reviewer 1 and the Academic Editor, and that it is an improved manuscript.

Thank you for the opportunity to revise this work and prepare it for publication.

Sincerely,

Greta Wengert

**Figure 1:**

>Cities: from Populated Places, US Dept of transportation Bureau of Transportation Statistics,
<https://data-usdot.opendata.arcgis.com/datasets/populated-places?geometry=-125.980%2C28.631%2C128.200%2C67.075>
There are no access and use constraints

>States: from TIGER <https://www.census.gov/geographies/mapping-files/time-series/geo/carto-boundary-file.html>

Copyright protection is not available for any work of the United States Government (Title 17 U.S.C., Section 105).

<https://www2.census.gov/geo/pdfs/maps-data/data/tiger/tgrshp2019/TGRSHP2019_TechDoc.pdf>

>Hillshade: 3DEP Elevation Hillshade from USGS National Map <https://www.usgs.gov/news/new-elevation-map-service-available-usgs-3d-elevation-program>

>Public lands: PAD US - no use constraints, in the public domain:

<https://databasin.org/datasets/df257819684049f9ad5f3b21a2a05e03/>
<https://databasin.org/datasets/99374897882a47499a0bc306fd4386b9/>
<https://databasin.org/datasets/fddafbb85f68418f8d9ff472d6ff25a8/>

>Our modeling extent

**Figure 2:**

>Cities: from Populated Places, US Dept of transportation Bureau of Transportation Statistics,
<https://data-usdot.opendata.arcgis.com/datasets/populated-places?geometry=-125.980%2C28.631%2C128.200%2C67.075>
There are no access and use constraints

>States: from TIGER <https://www.census.gov/geographies/mapping-files/time-series/geo/carto-boundary-file.html>

Copyright protection is not available for any work of the United States Government (Title 17 U.S.C., Section 105).

<https://www2.census.gov/geo/pdfs/maps-data/data/tiger/tgrshp2019/TGRSHP2019_TechDoc.pdf>

>Hillshade: 3DEP Elevation Hillshade from USGS National Map <https://www.usgs.gov/news/new-elevation-map-service-available-usgs-3d-elevation-program>

>Our modeling extent

>Our model output

**Figure 3:**

>Cities: from Populated Places, US Dept of transportation Bureau of Transportation Statistics,
<https://data-usdot.opendata.arcgis.com/datasets/populated-places?geometry=-125.980%2C28.631%2C128.200%2C67.075>
There are no access and use constraints

>States: from TIGER <https://www.census.gov/geographies/mapping-files/time-series/geo/carto-boundary-file.html>

Copyright protection is not available for any work of the United States Government (Title 17 U.S.C., Section 105).

<https://www2.census.gov/geo/pdfs/maps-data/data/tiger/tgrshp2019/TGRSHP2019_TechDoc.pdf>

>Hillshade: 3DEP Elevation Hillshade from USGS National Map <https://www.usgs.gov/news/new-elevation-map-service-available-usgs-3d-elevation-program>

>Our modeling extent

>Our model output summarized by Hydrologic Units: Watershed Boundary Dataset (WBD) <https://apps.nationalmap.gov/datasets/>

**Figure 5:**

>States: from TIGER <https://www.census.gov/geographies/mapping-files/time-series/geo/carto-boundary-file.html>

Copyright protection is not available for any work of the United States Government (Title 17 U.S.C., Section 105).

<https://www2.census.gov/geo/pdfs/maps-data/data/tiger/tgrshp2019/TGRSHP2019_TechDoc.pdf>

>Hillshade: 3DEP Elevation Hillshade from USGS National Map <https://www.usgs.gov/news/new-elevation-map-service-available-usgs-3d-elevation-program>

>Our modeling extent

>Our model output

>Fisher habitat: US Fish and Wildlife Service 2015. We have this up on Data Basin under [Creative Commons Attribution 3.0 License](https://creativecommons.org/licenses/by/3.0).

<https://databasin.org/datasets/38c1bc11ec4f4f8a935e7163ddfae58d/>

>NSO habitat: Davis et al. 2016
>Humboldt marten habitat: Schrott and Shinn 2020
